# Genomic Profiling Reveals Clinically Relevant Antimicrobial Resistance and Virulence Genes in *Klebsiella pneumoniae* from Hong Kong Wet Markets

**DOI:** 10.3390/antibiotics14090922

**Published:** 2025-09-12

**Authors:** Wing Yui Ngan, Subramanya Rao, Aster Hei Yiu Fung, Olivier Habimana

**Affiliations:** 1 Department of Pharmacology, University of Cambridge, Cambridge CB2 1PD, UK; 2 The School of Biological Sciences, The University of Hong Kong, Pokfulam, Hong Kong SAR, China; subbu36@gmail.com (S.R.); aster.fung@ha.org.hk (A.H.Y.F.); 3 Biotechnology and Food Engineering Program, Guangdong Technion-Israel Institute of Technology, Shantou 515063, China; 4 Faculty of Biotechnology and Food Engineering, Technion-Israel Institute of Technology, Haifa 3200003, Israel

**Keywords:** *Klebsiella pneumoniae*, antimicrobial resistance, virulence factors, wet market, One Health, biofilm, horizontal gene transfer

## Abstract

**Background:***Klebsiella pneumoniae* is a highly dangerous microorganism that presents significant challenges to effectively eliminate in food production facilities, making it a serious and urgent public health concern. The wet markets of Hong Kong represent a considerable yet insufficiently explored source for the spread of microorganisms. **Methods:** This investigation employed whole-genome sequencing and comparative genomics to assess the genomic variation and adaptive traits of *K. pneumoniae* extracted from wooden cutting boards in these marketplaces. We examined four wet market isolates in conjunction with 39 publicly accessible genomes from diverse origins. **Results:** Pan-genome analysis revealed a diverse and open genetic structure significantly shaped by horizontal gene transfer. Phylogenetic reconstruction did not categorize the wet market isolates into a singular clade, indicating varied contamination sources; nonetheless, certain market isolates exhibited close phylogenetic affiliations with high-risk clinical clones, implying possible spillover events. These isolates exhibited a concerning variety of antimicrobial resistance genes (ARGs), chiefly encoding efflux pumps (acrAB, oqxAB), which confer resistance to numerous drug categories. Moreover, the evaluation for pathogenicity attributes uncovered genes associated with robust biofilm development (fim and mrk operons) and efficient iron procurement strategies. **Conclusions:** The existence of these genetically adaptable isolates, possessing multidrug resistance and virulence factors, renders wet markets potential amplifiers and reservoirs for the spread of resistant pathogens. These findings present the initial genomic evidence of such risks in Hong Kong’s wet markets and emphasize the immediate necessity for improved hygiene protocols and comprehensive One Health surveillance to reduce transmission at the human–animal–environment interface.

## 1. Introduction

*Klebsiella pneumoniae* is an opportunistic pathogen with notable antimicrobial resistance and adaptability in different environments, emphasizing the need to understand its transmission and survival, particularly in food processing settings [1,2]. Wet markets are prevalent locations globally where individuals purchase fresh produce. They are large and not well-studied places for multidrug-resistant microorganisms to grow and live [3,4]. A combination of factors, such as traditional practices, a wide variety of food products, and poor sanitation, makes it easy for strong microbial communities to grow [5]. The continued reliance on wooden cutting boards across a variety of commercial sectors, especially in developing areas, intensifies this risk [6]. Their porous nature complicates cleaning, allowing bacteria infiltration and organic accumulation in minute fissures [6]. Bacteria serve as a constant nutrient source that enhances biofilm development and stability [4,6]. Consequently, biofilms are recognized to assist bacteria in enduring against pressures such as sanitizers and antimicrobial substances [7].

Biofilms on substrates that engage with alimentary substances, such as these timber boards, constitute a significant mechanism through which cross-contamination occurs [8]. Pathogens embedded within these matrices can easily move to food, which directly puts food safety at risk and could cause foodborne illness outbreaks. The high number of people shopping and the fact that wet markets are close to other sources of contamination make this public health threat even worse [9]. Although many global wet markets have made improvements to hygiene, it is still not clear how well these changes work or how well they are measured.

In Hong Kong, considerable challenges with facilities and hygiene endure in wet markets despite efforts towards modernization [10]. Notwithstanding the above acknowledged difficulties, the enforcement and effectiveness of local hygiene ordinances, including the Food Business Regulation (Cap. 132X) on the cleanliness of market shelves, have not been adequately tested as specific inhibitors of foodborne pathogenic biofilms (https://www.elegislation.gov.hk/hk/cap132X!en-zh-Hant-HK) (accessed on 7 August 2025) [6,9]. A lot of markets still do not have the tools they need to store, manage, and throw away food properly, and there is not enough information about how well current rules prevent microbial contamination on surfaces like wooden cutting boards. Prior studies indicate that the cleaning protocols in Hong Kong’s wet markets are insufficient for controlling pathogenic microorganisms, including *K. pneumoniae* [9]. Nonetheless, the exact origins of this contamination and the genetic mechanisms that enable its persistence and resistance in these environments remain predominantly unidentified [4].

We must address these knowledge gaps to develop effective interventions to reduce transmission risk. To address three key questions, genomic insights are essential: First, what are the differences between *K. pneumoniae* genomes from Hong Kong wet markets and those from clinical and environmental settings? Second, do evolutionary relationship assessments reveal proof of cross-contamination and the spread of resistant clones among healthcare, ecological, and food-manufacturing environments? Third, do these wet marketplace isolates exhibit distinct genetic characteristics—such as enhanced biofilm development or a wider spectrum of antimicrobial resistance genes—that facilitate their endurance in these challenging surroundings?

We suggest that wet market isolates are a genetically varied population shaped by horizontal gene transfer (HGT), showing genomic traits that reflect adaptation to ecological and human-induced stresses, such as disinfectants and antimicrobial agents [11]. This research addresses these critical issues through an extensive genomic analysis of *K. pneumoniae* isolates, which were systematically collected from various wet markets in the lively and densely populated locale of Hong Kong. We employed whole-genome sequencing in conjunction with comparative genomic analyses to investigate four strains that had been previously isolated, comparing them to a comprehensive array of publicly available genomic data. Our investigations were primarily focused on the identification of genetic indicators correlated with pathogenicity and, of paramount significance, with profiles of antimicrobial resistance. This study seeks to clarify the function of wet markets as potential amplifiers of antimicrobial resistance by synthesizing data on AMR genes, virulence factors, and phylogenetic relationships, thereby informing more effective public health strategies and interventions against this widespread pathogen.

## 2. Materials and Methods

### 2.1. Strain Selection and Genome Sequencing

The four *K. pneumoniae* variants examined in this research were obtained from wooden cutting boards in Hong Kong wet markets during a previous environmental sampling initiative [9]. Sampling targeted high-contact areas (10 cm^2^) of pork-processing cutting boards using pre-sterilized cotton swabs hydrated with sterile PBS. To account for the porous surface topography, a combined swabbing–friction technique was employed, wherein the swabs were rigorously scrubbed across the cutting boards to maximize microbial recovery. The swabs were promptly placed in Amies Charcoal medium and kept cool during transit to the laboratory.

The swab samples were enriched in TSB at 30 °C for 20 h and then plated onto TSA. After a 20 h incubation at 30 °C, the colonies were transferred to TSB for the purpose of obtaining pure isolates. Genomic DNA was extracted from monocultures with the PureLink Microbiome DNA Purification Kit (ThermoFisher Scientific, Waltham, MA, USA), and DNA quality was checked using BioDrop DUO spectrophotometry (BioDrop, Cambridge, UK). Isolates were identified as *K. pneumoniae* through metagenomic 16S V3-V4 sequencing (Illumina HiSeq PE250 platform at Novogene Co., Beijing, China) and analysis via the Parallel-Meta pipeline [12], with sequence data deposited in the European Nucleotide Archive (PRJEB33545).

For the purpose of comparative analysis, 39 publicly accessible *K. pneumoniae* genomes were carefully chosen from NCBI GenBank to illustrate a wide variety of ecological niches. The public genomes were categorized as follows: 13 clinical, 12 environmental, and 14 animal-associated (further specified in Table 1). This selection criterion prioritized the following: (1) assembly completeness (≥95% BUSCO scores), (2) balanced representation of major niches (13 clinical, 12 environmental, 14 animal-associated, with animal isolates further specified in Table 1), and (3) inclusion of globally prevalent sequence types (e.g., ST258, ST11, ST307). Genomes with ambiguous metadata or fragmented assemblies were excluded to ensure robust comparative analyses.

### 2.2. Genome Assembly, Annotation, and Quality Control

Raw reads underwent quality control with FastQC 0.11.7, followed by adapter trimming and filtering for a minimum read length of 36 base pairs and quality score of 15 using Trimmomatic v0.36 [13]. De novo genomic assembly was conducted employing SPAdes v3.11.176 [14] with default settings. Genome annotation utilized Prokka v1.7 [15], with the GenBank compliance flag, ensuring standardized gene calls. To mitigate annotation bias, *K. pneumoniae* retrieved from public databases were re-annotated with Prokka using identical parameters. Genome completeness was assessed using contig ordering and alignment leveraged MAUVE [16], with final genome concatenation accomplished using Artemis [17]. Insertion sequences were identified using ISsaga [18], genomic islands using IslandViewer 4 [19], and multilocus sequence types (MLSTs) assigned using the pubMLST database [20].

### 2.3. Comparative Genomics and Phylogenetic Analysis

Pan-genome analysis was conducted using Roary v3.13.0 with Prokka-generated GFF3 files [21]. Default settings were employed (95% amino acid identity threshold for ortholog detection). Fundamental genes were delineated as existing in ≥99% of the genomic sequences, whereas supplementary genes included the residual gene assortment. Unique genes were identified using the Roary “gene_presence_absence.csv” output file. This initial pangenome analysis was further refined using Pan-Explorer [22], focusing on the identification and characterization of accessory genes. Pan-Explorer’s output provided valuable information regarding the distribution of accessory genes among different sample types. Subsequently, the core-genome phylogeny was constructed using a concatenated alignment of the core genes identified via Roary, employing RAxML v8.2.12 [23] with the GTRGAMMA model and 100 bootstrap replicates for assessing branch support. Tree visualization was performed using iTOL v6 [24].

To further investigate the evolutionary dynamics of the accessory gene content, TORMES was used [25]. TORMES facilitated the identification of clusters of orthologous genes (COGs) found within the accessory genome, helping to characterize groups of genes potentially acquired through horizontal gene transfer events. The accessory genes were categorized into different groups based on their functional characteristics as identified by TORMES. The results of this analysis informed the selection of specific genes for further investigation (cf. Results Section 3.6), which show phylogenies for three genes chosen because of distinct evolutionary trajectories in the TORMES results; note that these figures are visual representations of these evolutionary dynamics among multiple samples). Four accessory genes (*ycgR*, *sasA*, *bamA*, and *fimD*) were selected for phylogenetic and synteny analysis due to their distinct evolutionary trajectories indicated in the preliminary TORMES analysis and their potential roles in significant biological processes: *ycgR* (motility regulation), *sasA* (response to stress), *bamA* (development of the outer membrane), and *fimD* (biofilm formation via fimbrial assembly). The phylogenetic analysis of the genes *ycgR*, *sasA*, *bamA*, and *fimD* was performed using RAxML, as described previously.

A phylogenetic assessment was performed utilizing 43 genomes, which included four isolates from wet markets alongside 39 genomes sourced from public databases. Whole-genome alignment was performed with Parsnp v1.2 (Harvest Suite) [26]. This generated a maximum likelihood phylogenetic tree based on core-genome single nucleotide polymorphisms (SNPs), after excluding recombinatorial regions identified during the Parsnp analysis. The resulting phylogeny complemented the core-genome phylogeny obtained from Roary and provided additional insights into the evolutionary relationships among the isolates.

### 2.4. Variant Calling and Functional Analysis

Paired-end reads were aligned with the *Klebsiella pneumoniae* ATCC genome using Bowtie2 2.3.2 [27]. SNPs and indels were identified through SAMtools 1.7 [28], with a quality score threshold of 50. Moreover, SnpEff version 4.3 was employed for variant annotation, forecasting their possible functional consequences [29]. This study provided an extensive examination of the genetic diversity present among the isolates.

### 2.5. Antibiotic Resistance and Virulence Gene Detection

The annotated gbk files from Prokka were screened for antibiotic resistance genes and virulence factors using ABRicate (https://github.com/tseemann/abricate) (accessed on 7 August 2025). The built-in ResFinder 4.1 database was employed to identify acquired resistance genes [30]. Moreover, the Comprehensive Antibiotic Resistance Database (CARD) (https://card.mcmaster.ca) and ARG-ANNOT [31] were employed to meticulously curate the list of genes. This approach ensured the identification of any resistance genes missed by ResFinder, including those with non-synonymous SNPs.

The existence of pathogenicity determinants was ascertained by evaluating chosen genomic sequences against the Virulence Factor Database (VFDB). The updated VFDB was downloaded from www.mgc.ac.cn/ on 23 September 2024. The results were organized utilizing Python, and heatmaps were produced using R environment (version 4.0.3).

### 2.6. Statistical Analysis

PCA was performed using R (https://www.R-project.org) to show genetic diversity among the isolates. Network analysis of accessory gene clusters, including the calculation of network properties (e.g., clustering coefficients, betweenness centrality, and modularity), was performed using the Pan-Explorer pipeline [22] to uncover potential horizontal gene transfer patterns. This helped in understanding the spread of acquired genes within the collection of the samples investigated. The results derived from these statistical analyses were utilized to evaluate the statistical significance of any observed associations between genetic characteristics and the sample origin. Specific statistical tests (e.g., likelihood ratio tests) were applied as appropriate, indicated in the corresponding result sections.

## 3. Results

### 3.1. Phylogenetic Analysis

**Figure 1:**
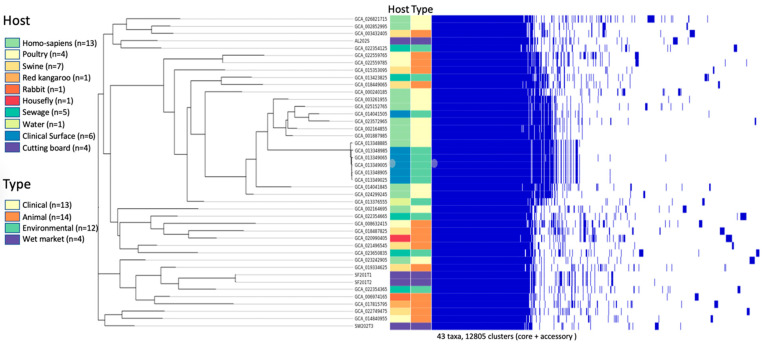
A phylogenetic tree based on core-genome SNPs that shows the most likely relationships. (**Left panel**) There are 43 K. pneumoniae isolates in the tree: 4 from Hong Kong wet markets (marked in red) and 39 from public databases. The color of the tips depends on where the sample came from. Key nodes indicate bootstrap support ratings of 80% or higher. (**Right Panel**) A clustered heatmap of the accessory genome, representing 12,805 genomic clusters. The presence of a gene cluster in an isolate is shown in blue, and absence is shown in white. The dendrogram represents hierarchical clustering based on gene content similarity, illustrating the relatedness of isolates based on their accessory genome.

Core-genome SNP phylogeny (Figure 1) split the wet market isolates into two groups: AL202S and SW202T3 clustered with clinical ST258 strains (bootstrap = 92%), whereas SF201T1/SF201T2 formed a separate branch near environmental ST307 (bootstrap = 85%). This bifurcation suggests multiple contamination sources for wet market *K. pneumoniae*.

Human and clinical isolates form relatively tight clusters, indicative of close genetic relationships. This uniformity implies restricted genetic variability among clinical isolates, potentially resulting from localized spread or shared hospital-associated sources. Animal isolates, especially those identified as poultry and swine isolates, are grouped into moderately tight clusters, suggesting interspecies transmission within agricultural settings. Notably, rabbit and housefly isolates are more dispersed, indicating higher genetic diversity or the influence of distinct ecological niches. Environmental and surface isolates demonstrate broader clustering, especially those from sewage and water, reflecting high genetic diversity. This diversity likely arises from the wide range of potential environmental sources and transmission pathways.

The heatmap identified 12,805 genomic clusters, emphasizing core and accessory genome components. Core clusters exhibit limited variation and are prevalent across most isolates, whereas accessory clusters show greater variability. Environmental and animal isolates exhibit significant heterogeneity in accessory genome content, suggesting adaptation to varying environmental pressures and host environments. Particular attention is drawn to the isolates from wet markets, which form distinct but loose clusters. These isolates exhibit substantial genomic heterogeneity, as highlighted by the heatmap, underscoring the dynamic interchange of genetic material likely facilitated by the diverse microbial and ecological milieu of wet market environments. This heterogeneity is in accordance with the theory that wet markets function as hubs for microbial interchange and development, possibly accelerating the emergence of novel pathogenic variants.

The presented results highlight the variability influenced by host and environmental contexts, with wet market isolates demonstrating significant genetic diversity and adaptability. These findings suggest that to manage *Klebsiella pneumoniae* spread within ecosystems, continuous monitoring and genetic examination are essential.

The core-genome phylogeny-derived population structure was subsequently corroborated through a multivariate analysis of the accessory genome. Principal Component Analysis (PCA) of gene presence/absence patterns offered an additional statistical insight into the genetic affiliations among isolates from various niches.

### 3.2. Principal Component Analysis (PCA)

**Figure 2:**
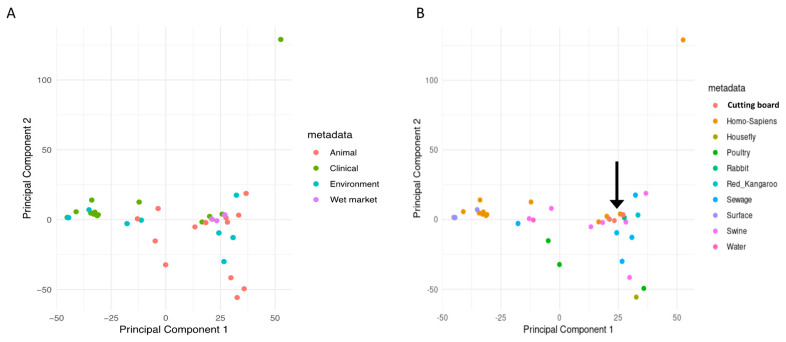
Principal Component Analysis (PCA) showing patterns of gene presence and absence. (**A**) A PCA plot that shows isolates by their wide ecological niche, such as animal, clinical, environment, or wet market. (**B**) PCA map with isolates colored by specific sample source, showing more detailed grouping and possible transmission routes (the arrow shows where the cutting board, animal, and sewage isolates overlap).

The PCA of gene presence/absence (Figure 2A) revealed a notable distinction among the niches (PERMANOVA, *p* = 0.003). Wet market isolates were between the clinical (PC1 = +0.62 ± 0.11) and environmental (PC1 = −0.38 ± 0.09) categories. SF201T2 showed particular proximity to sewage isolates, suggesting potential environmental transmission routes. A PCA was performed on 43 *Klebsiella pneumoniae* isolates from various sources to explore correlations between their genetic profiles and origins. Two distinct PCA plots are presented (Figure 2A,B). Figure 2A depicts the PCA based on sample type categories (animal, clinical, environment, wet market), while Figure 2B shows the PCA based on more specific sample source classifications (cutting board, Homo sapiens, housefly, poultry, rabbit, red kangaroo, sewage, surface, swine, water).

Figure 2A reveals a degree of clustering according to sample type. While the separation is not absolute, isolates from clinical sources and those from animal sources tend to cluster somewhat separately from environmental isolates. A notable observation is the distinct clustering of *K. pneumoniae* isolates from wet markets. These isolates are distinct on the PCA plot from clinical and animal isolates, with some overlap. This suggests that wet market *K. pneumoniae* strains may possess distinct genomic features compared to those from other sample types. This warrants further investigation into the genetic mechanisms underlying this differentiation. The relatively wide spread of the ‘animal’ samples suggests significant genetic heterogeneity among *K. pneumoniae* isolates from animal sources, which could be attributed to numerous factors, including host species, geographical locations, and exposure to various environmental stressors.

Figure 2B, with its finer-grained source classification, provides more detail. The grouping arrangement is intricate, and isolates display varying degrees of aggregation. A particularly striking observation is the clear separation of the isolates sourced from cutting boards from those found in other sources, such as swine and sewage. While both groups of isolates partially overlap, the presence of a distinct cutting board cluster highlights the potential for these surfaces to harbor unique *K. pneumoniae* populations. The arrow in Figure 2B points to a region of overlap between isolates from cutting boards, sewage, and various animal sources. This overlap suggests a potential source–sink dynamic. Possible routes of transmission from animal sources through sewage to cutting boards are suggested by the proximity of the points in the PCA plot. This overlap warrants further epidemiological investigation to confirm possible transmission routes. The clear separation of *K. pneumoniae* isolates from humans and those from other sources, notably the animal and environmental isolates, also requires further exploration. The overall pattern in both Figure 2A,B suggests a genetic diversity within *K. pneumoniae* populations that is strongly influenced by their host and environmental origin.

Subsequent analyses, which encompass phylogenetic reconstruction and comprehensive genomic comparison—such as the application of core-genome multilocus sequence typing or whole-genome sequencing—are imperative for elucidating the genetic factors that contribute to the observed clustering patterns. These examinations aim to gain insights into the evolutionary dynamics and transmission pathways in these populations. The data presented here suggest that wet markets and cutting boards might act as reservoirs or hotspots of *K. pneumoniae* genetic diversity and transmission, particularly concerning the potential for zoonotic spread and subsequent human infection. This aspect requires additional detailed investigation.

Upon reviewing demographic data and ecological classifications, we sought to articulate the essential genetic attributes that facilitate this diversity, guaranteeing a meticulous and well-founded analysis. For this purpose, we conducted a pan-genome examination to clarify the fundamental and supplementary elements that characterize the genetic inventory of this demographic.

### 3.3. Pan-Genome Analysis of 43 K. pneumoniae Genomes

**Figure 3:**
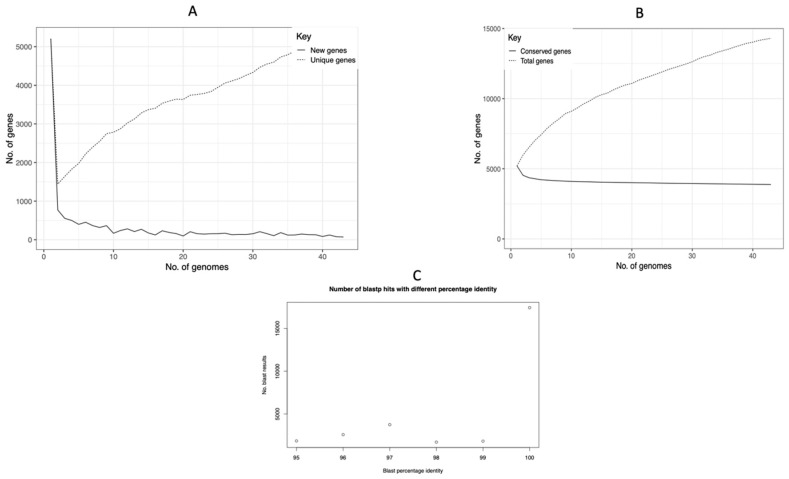
A pan-genome study of 43 *Klebsiella pneumoniae* genomes. (**A**) A graph illustrating the quantity of core genes (present in a minimum of 99% of genomic sequences) as a variable of the quantity of genomes incorporated. (**B**) Pan-genome trajectory illustrating the cumulative quantity of genes (core + accessory) as genomes are incorporated sequentially. This shows that the pan-genome is open. (**C**) Distribution of BLAST hit identity percentages for all gene sequences, showing that the genomes are very different from one another.

We analyzed 43 *K. pneumoniae* genomes, including 4 from wet markets and 39 publicly available, using Roary with a 95% amino acid identity threshold. This examination identified 12,805 genes, classified as core (occurring in ≥99% of genomic sequences), supplementary, and strain-specific components. The core genome plateaued at approximately 4000 genes (Figure 3A), indicating comprehensive sampling of conserved functions, while accessory genes continued to accumulate with each added genome (slope = 12.4 genes/genome), revealing an open pan-genome structure characteristic of extensive horizontal gene transfer potential. This pattern was further supported by the cumulative gene curves (Figure 3B) and the distribution of BLAST hits (ncbi blast+ 2.6.0) (Figure 3C), which showed limited high-identity matches (≥98%), consistent with substantial genomic variability.

**Figure 4:**
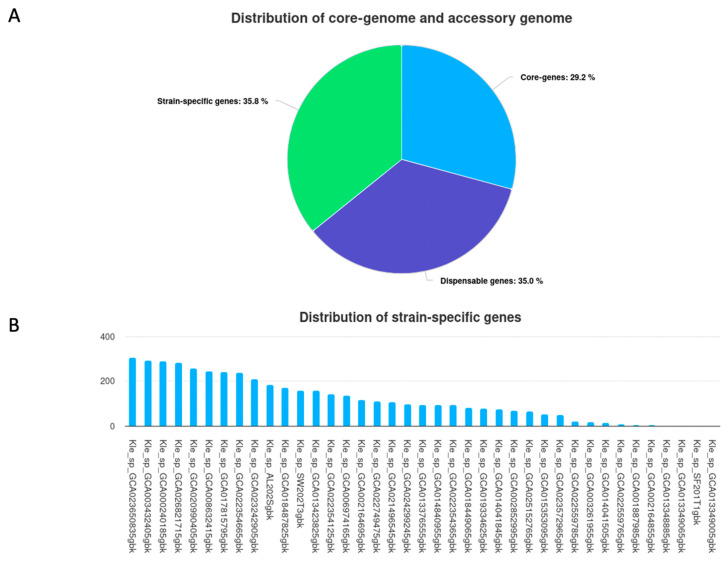
The parts that make up the *Klebsiella pneumoniae* pan-genome. (**A**) A pie chart that shows the distribution of core, accessory, and strain-specific genes across the 43 genomes. (**B**) A bar graph that illustrates the quantity of distinct (strain-specific) genes identified in each of the four wet market isolates (AL202S, SW202T3, SF201T1, and SF201T2).

The pan-genome encompassed 29.2% core (3742 genes), 35.0% supplementary (4482 genes), and 35.8% strain-specific (4581 genes) components (Figure 4A). Notably, wet market isolates contributed 344 strain-specific genes (7.5% of total), with AL202S harboring the most (183 genes), including putative efflux pumps (*acrB*, *oqxB*) and biofilm regulators (*bssS*), followed by SW202T3 (159 genes) and SF201T1 (2 genes). Conversely, SF201T2 exhibited an absence of unique genes, indicating a recent introduction from a less diverse lineage (Figure 4B). This distribution underscores the significant genetic variation among the species and the specific adaptations of wet market isolates to their ecological context.

Our investigation identified diverse antimicrobial resistance genes (ARGs) within isolates obtained from the wet market. This pan-genome encompasses genes that enhance adaptive characteristics. Consequently, we meticulously examined this adaptable genomic reservoir for factors affecting antimicrobial resistance, a critical public health concern.

### 3.4. Antimicrobial Resistance Genes

**Figure 5:**
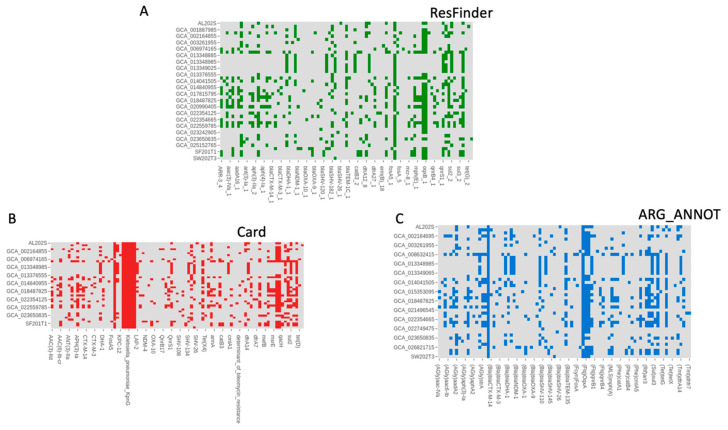
Heatmaps showing the antimicrobial resistance genes (ARGs) found in the 43 *K. pneumoniae* isolates. We employed (**A**) ResFinder, (**B**) the Comprehensive Antibiotic Resistance Database (CARD), and (**C**) ARG-ANNOT for the identification of ARGs. Rows show individual isolates (wet market isolates are marked), while columns show resistance genes. A filled box shows that a gene is present. The isolates are categorized by their sources. Isolates without detected ARGs for a given database are not represented by a row in the respective heatmap.

Regarding antimicrobial resistance genes and virulence factors, ResFinder analysis (Figure 5A) indicated a variety of ARGs among the isolates, including those from the wet market. Notably, AL202S had the highest count of ARGs. Both CARD and ARG_ANNOT (Figure 5B,C) detected ARGs that provide resistance to multiple antibiotic classes. Wet market isolates presented a distinct pattern of resistance genes compared to those from clinical and environmental sources. The provided tables detail the antibiotic resistance genes (ARGs) identified in horizontal gene transfer regions from four samples: AL202S, SF201T1, SF201T2, and SW202T3. The data indicate a wide variety of ARGs that provide resistance against a large range of antibiotics. Antibiotic expulsion, which is facilitated by various expulsion pump families, encompassing ATP-binding cassette (ABC) transporters, Major Facilitator Superfamily (MFS), and Resistance–Nodulation–Cell Division (RND), appears to be the principal mechanism of resistance. Additionally, some ARGs modify antibiotic targets, thereby reducing their effectiveness.

The most prevalent mechanism is antibiotic efflux, particularly via RND pumps (*acrA*, *acrB*) present in all four samples. MFS pumps (*KpnE*, *KpnF*, *KpnG*, *KpnH*, *tet(A)*, *tet(E)*) are also frequently observed, showcasing the significant role of efflux in multidrug resistance. ABC transporters (*msbA*) contribute to resistance in several samples. Several ARGs modify the antibiotic target site. Examples include *eptB* (which modifies the peptidic antibiotic target) and mutations in *EF-Tu* (which alter elfamycin binding), found in sample SW202T3. Mutations in genes such as *ompA*, which affect outer membrane permeability, are also observed, reducing antibiotic uptake. *FosA6*, identified in samples SF201T2 and SW202T3, represents an example of an enzyme responsible for antibiotic inactivation. Each sample exhibits a unique ARG profile. Sample AL202 displays a greater diversity of ARGs and associated resistance mechanisms than the others. Samples SF201T1, SF201T2, and SW202T3 show overlap in some ARGs (e.g., *acrB*, *msbA*, *tet(E*), and *ompA*), but each also possesses unique ARGs, suggesting diverse acquisition pathways. *acrB* is consistently present across all samples, indicating a potentially common ancestral source or horizontal transfer event involving this crucial RND efflux pump. Other shared ARGs include *ompA*, *msbA,* and tetracycline resistance genes (*tet(A*), *tet(E*)). Sample AL202S distinguishes itself with a wider range of ARGs involved in resistance to various drug classes. Samples SF201T1, SF201T2, and SW202T3 have a more focused set of resistance genes, implying different selective pressures or acquisition events.

### 3.5. Comparative Genomic Analysis of Virulence Factors in Klebsiella pneumoniae Isolates from Wet Market Samples

Pathogenic efficacy depends on virulence strategies that facilitate host colonization and endurance while concurrently evading antimicrobials. Consequently, we augmented our resistance gene characterization with a comprehensive examination for virulence determinants to assess the complete pathogenic capacity of these isolates.

**Figure 6:**
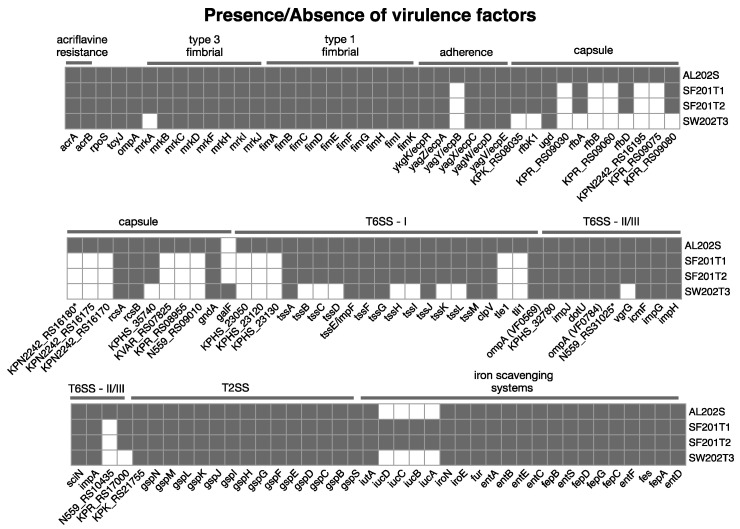
A heatmap showing the virulence factors found in the four *K. pneumoniae* isolates from wet markets. We used the Virulence Factor Database (VFDB) to find genes. The four isolates are represented in the columns, while the rows illustrate virulence genes classified by their functionality (for example, fimbriae, secretion mechanisms, and iron acquisition). A filled box shows that a gene is present. The asterisk (*) next to the gene identifiers KPN2242 (locus tag RS16180) and N559 (locus tag RS31025) denotes that these specific genes were annotated with general functional names by Prokka but were manually re-annotated as putative virulence factors based on lower-confidence homology matches to the VFDB, distinguishing them from genes with high-confidence hits or canonical virulence factor annotations.

The virulence factor profiles of four *Klebsiella pneumoniae* isolates (AL202S, SF201T1, SF201T2, and SW202T3) utilizing the VFDB database are presented in Figure 6.

All isolates exhibited the entirety of the *fimABCDEFGHIK* operon, which encodes type 1 fimbriae, an essential element in the process of biofilm development. Three isolates (AL202S, SF201T1, and SF201T2) also harbored the *mrkABCDFHIJ* genes associated with type 3 fimbriae production; SW202T3 lacked *mrkA* but retained the remaining genes. The *ecpABCDER* gene cluster, involved in biofilm formation, was complete in AL202S but partially incomplete (missing *ecpB*) in SF201T1, SF201T2, and SW202T3. This suggests potential variations in biofilm production capacity among the isolates.

Wet market *K. pneumoniae* isolates exhibited genes associated with the type II secretion system (T2SS; *gspC-M*, *gspB*, *gspN*, *gspS*). Three isolates (AL202S, SF201T1, and SF201T2) possessed a complete T6SS-I gene cluster (*tssA-M*), while SW202T3 showed a less complete profile, possessing several individual T6SS-related genes (*clpV*, *tle1*, *tli1*, *imp*, etc.) but lacking the complete cluster. This suggests variations in the functionality and potential of the T6SS among isolates.

All four isolates contained genes involved in iron scavenging, including *iutA*, *iroNE*, *entABCDEF*, *Fur*, *fepABCDG,* and *Fes*. The *iucABCD* operon, implicated in aerobactin biosynthesis, was detectable solely in SF201T1 and SF201T2. This variation suggests potential differences in iron acquisition strategies.

The isolates demonstrated varied genetic profiles (*rfb*, *rcs*, *gndA*, etc.) linked to capsule biosynthesis. Diversity in capsular polysaccharide architecture may influence immune evasion and survival outcomes.

AL202S contained the most comprehensive set of virulence factors, while SW202T3 exhibited a less extensive profile. The similarities between SF201T1 and SF201T2 indicate a likely shared origin or comparable selective influences. Conversely, the unique virulence factor profile of SW202T3 suggests a distinct origin and potentially different environmental adaptations.

Horizontal gene transfer is vital for pathogen evolution by integrating virulence and resistance genes. We selected key accessory genes for detailed analysis to understand their acquisition and explore the development of adaptive traits.

### 3.6. Phylogenetic and Genomic Contextualization of Selected Accessory Genes in Klebsiella Isolates

**Figure 7:**
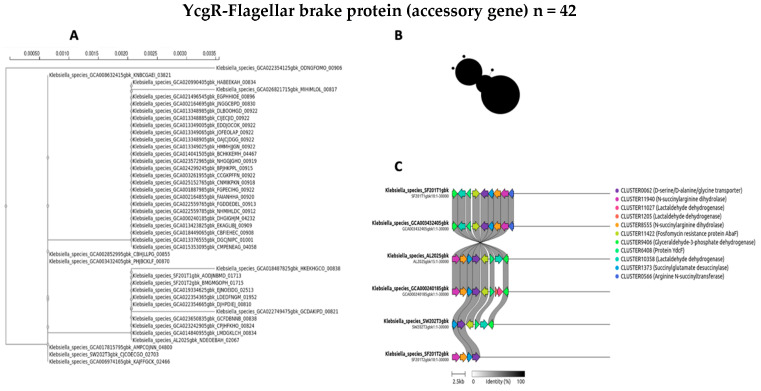
Evolutionary study of the accessory gene *ycgR* (flagellar brake protein). (**A**) The *ycgR* gene sequence’s maximum likelihood phylogenetic tree. (**B**) Network of co-occurrence for *ycgR,* where *ycgR* genes are found together in different genomes. Node size is proportional to the number of shared accessory genes with other nodes in the network. Edges connect genomes that contain the *ycgR* gene. (**C**) Synteny plots of the genomic area surrounding *ycgR* in a selection of isolates, illustrating highly varied flanking regions.

**Figure 8:**
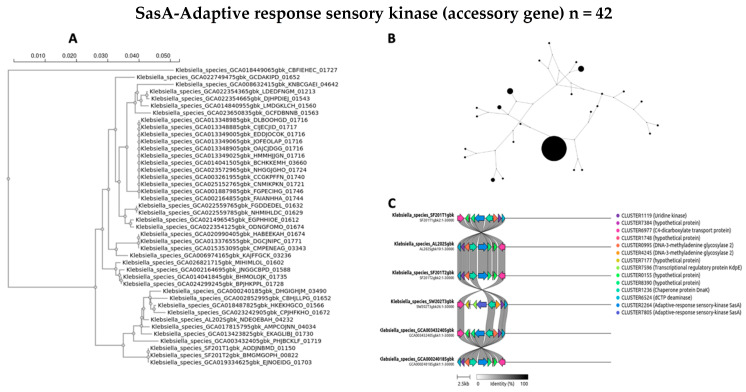
An evolutionary study of the accessory gene *sasA* (adaptive response sensory kinase). (**A**) The maximum likelihood phylogenetic tree of the *sasA* gene sequence. (**B**) Network of co-occurrence for *sasA.* Node size is proportional to the number of shared accessory genes with other nodes in the network. Edges connect genomes that contain the *sasA* gene. (**C**) Synteny plots of the *sasA* genomic region, demonstrating a moderate level of conservation in certain isolates.

**Figure 9:**
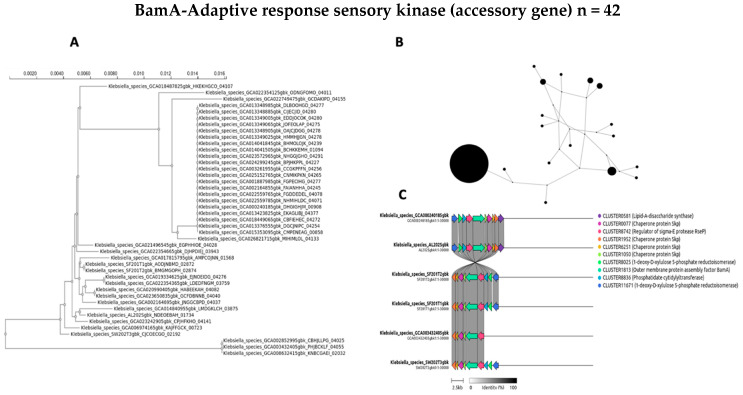
Evolutionary study of the *bamA* accessory gene (outer membrane protein assembly factor). (**A**) Maximum likelihood phylogenetic tree of the *bamA* gene sequence. (**B**) Network of co-occurrence for *bamA*. Node size is proportional to the number of shared accessory genes with other nodes in the network. Edges connect genomes that contain the *bamA* gene. (**C**) Synteny plots of the *bamA* genomic area, showing a mix of conservation and change.

**Figure 10:**
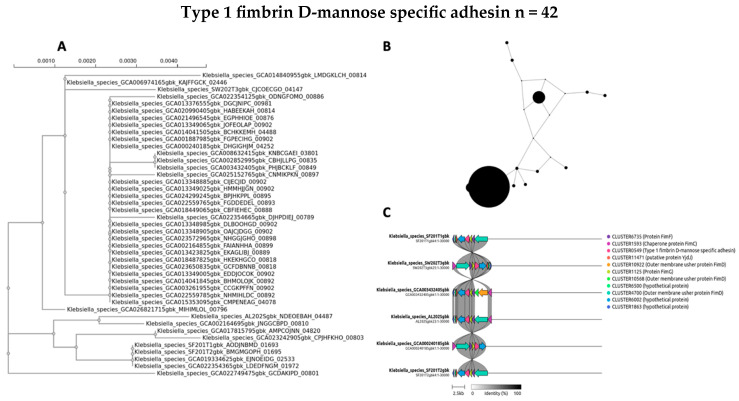
Evolutionary study of the accessory gene *fimD* (type 1 fimbrial adhesin). (**A**) Maximum likelihood phylogenetic tree of the *fimD* gene sequence. (**B**) Network of co-occurrence for *fimD*, which shows clusters that are very dense and well-connected. Node size is proportional to the number of shared accessory genes with other nodes in the network. Edges connect genomes that contain the *fimD* gene. (**C**) Synteny plots of the *fimD* genomic area show that most isolates have a very similar genetic background.

The evolutionary dynamics and genomic distribution of four accessory genes (*ycgR*, *sasA*, *bamA*, and *fimD*) were separately investigated using phylogenetic analysis (A), network visualization (B), and synteny assessment (C) (Figure 7, Figure 8, Figure 9 and Figure 10).

To investigate the evolutionary relationships and distribution of accessory genes across the diverse *Klebsiella* strains, phylogenetic trees for four selected genes were constructed for *ycgR*, *sasA*, *bamA*, and the type 1 fimbrin D-mannose-specific adhesin (Figure 7A, Figure 8A, Figure 9A and Figure 10A, respectively). These genes were chosen for their roles and presence in the accessory genome. The phylogenetic tree of the *ycgR* gene (Figure 7A), encoding a flagellar brake protein, reveals a complex pattern of relationships. Diverse degrees of sequence divergence are denoted by branch lengths. Some strains cluster tightly, suggesting recent common ancestry and potential horizontal gene transfer events. Notably, certain branches exhibit longer lengths, indicating substantial sequence divergence among strains. The lack of a clear, strong phylogenetic signal suggests that *ycgR* has experienced significant horizontal gene transfer or recombination, leading to a mosaic distribution among the studied *Klebsiella* isolates. The phylogenetic tree of *sasA* (Figure 8A), an adaptive response sensory kinase, shows a topology largely congruent with the overall *Klebsiella* phylogeny. This suggests a primarily vertical inheritance pattern for *sasA*, with limited evidence of recent widespread horizontal transfer. However, subtle incongruences between the *sasA* tree and the overall *Klebsiella* phylogeny remain, indicating some instances of possible horizontal gene transfer or recombination events that have shaped the evolution of *sasA* in certain lineages. The *bamA* phylogeny (Figure 9A) demonstrates a mixed pattern. Some clustering indicates vertical inheritance of this gene, similar to the *sasA* phylogeny. However, several long branches and instances of incongruence with expected *Klebsiella* phylogeny suggest that horizontal gene transfer has also played a role in the distribution and evolution of this gene. The presence of long branches suggests periods of accelerated evolution in some lineages, potentially driven by selective pressures. The phylogenetic analysis of the type 1 fimbrin D-mannose-specific adhesin gene (Figure 10A) exhibits a notable clustering structure. The tree reveals groups of closely related strains, suggesting potential for clonal expansion or frequent horizontal gene transfer within these specific groups. The phylogenetic dispersion of this adhesin gene seems to be more diverse in comparison to *sasA*, potentially indicating its participation in host–pathogen dynamics and acclimatization to various ecological environments.

A network analysis explored the accessory gene distribution in Klebsiella isolates. Figure 7B, Figure 8B, Figure 9B and Figure 10B depict networks for four selected accessory genes: *ycgR* (flagellar brake protein), *sasA* (adaptive response sensory kinase), *bamA* (outer membrane protein assembly factor), and the *fimD* gene (type 1 fimbrin D-mannose specific adhesin). Each node represents a *Klebsiella* genome, and each edge indicates the co-occurrence of the specific accessory gene in the two connected genomes. The node size is proportional to the number of shared accessory genes with other genomes within the network. The *ycgR* network (Figure 7B) exhibits a relatively dispersed structure lacking strongly defined clusters. This suggests independent acquisition via multiple horizontal gene transfer events rather than clonal transmission. The predominantly small node sizes reflect low overall genomic similarity among isolates possessing this gene. The *sasA* network (Figure 8B) displays increased connectivity compared to the *ycgR* network. While lacking dense, highly connected clusters, some denser groupings are visible, suggesting limited clonal association or increased likelihood of co-acquisition with other genes in this subset of isolates. The *bamA* network (Figure 9B) shows a topology similar to *sasA*, with moderate connectivity. This indicates an intermediate level of clonal relationship or co-acquisition, while the largest cluster hints at localized spread or shared ancestry. In contrast to the other genes, the *fimD* network (Figure 10B) displays prominent, densely connected clusters. This suggests substantial clonal expansion or highly localized horizontal gene transfer within specific isolate subgroups. The large node sizes are consistent with considerable shared genetic material among isolates possessing this gene. This suggests a potentially significant role for *fimD* in *Klebsiella* adaptation and transmission. The presence of prominent and dense clusters suggests a more extensive clonal expansion or strong horizontal gene transfer events within subsets of isolates. This gene could play a crucial role in the adjustment and dissemination of particular *Klebsiella* lineages.

To further elucidate the evolutionary history and potential acquisition mechanisms of the four selected accessory genes (*ycgR*, *sasA*, *bamA*, and *fimD*), we performed synteny analysis. Figure 7C, Figure 8C, Figure 9C and Figure 10C present the syntenic context of these genes within their respective genomic locations across the four wet market, one clinical, and one swine *Klebsiella* isolates. Each figure shows the genomic region around the target gene, highlighting gene order conservation and variations. Different colors represent different genes, with the target gene highlighted centrally. The presence or absence of flanking genes provides insight into the potential mode of acquisition (e.g., horizontal gene transfer via mobile genetic elements versus vertical inheritance). The *ycgR* synteny analysis (Figure 7C) reveals a highly variable genomic context. The absence of conserved flanking genes across two wet market *K. pneumoniae* isolates strongly suggests that *ycgR* acquisition occurred primarily through independent horizontal gene transfer events, likely mediated by mobile genetic elements such as plasmids or transposons. The diverse gene arrangements indicate that insertions occurred at different locations and contexts. The combination of a highly dispersed network topology and a complete lack of conserved synteny in the flanking genomic regions provides compelling evidence that the *ycgR* gene has been independently acquired on multiple occasions across different lineages. This pattern spreads through mobile genetic elements, not from a common ancestor. The synteny surrounding *sasA* (Figure 8C) shows some degree of conservation in the immediate vicinity of the gene in a subset of isolates, except for the SW202T3 wet market strain. A few isolates exhibit similar flanking genes, suggesting possible clonal relatedness or co-transfer events involving a small genomic island or a specific mobile element. However, significant variability is still present, implying independent horizontal transfer events as a significant contributor to *sasA* distribution. The moderate network connectivity, coupled with the partial conservation of its immediate syntenic context in a subset of isolates, points to a complex evolutionary history for *sasA*. This pattern suggests a foundation of vertical inheritance within certain clades, upon which subsequent horizontal gene transfer events have acted to distribute this sensory kinase gene into other, unrelated genetic backgrounds. The *bamA* synteny (Figure 9C) demonstrates a moderate level of conservation in the immediate flanking regions. The presence of several isolates sharing a similar gene arrangement in proximity to *bamA* suggests a certain degree of clonal inheritance or co-acquisition via the transfer of larger genomic fragments. Nonetheless, the noted variation suggests that horizontal gene transfer continued to influence the general distribution of *bamA*. The intermediate patterns observed in both the network analysis and the synteny plot for *bamA* indicate that its evolutionary history is not easily categorized. The evidence suggests a significant role for vertical inheritance in its propagation, but the presence of incongruent phylogenetic signals and variable genomic contexts also implies that horizontal transfer has contributed to the distribution of this essential outer membrane assembly factor among diverse strains. The synteny analysis for *fimD* (Figure 10C) reveals a strikingly higher level of conservation among isolates compared to other genes. In a significant number of isolates, a highly conserved gene order is observed in the vicinity of *fimD*, strongly indicating its acquisition and maintenance via vertical inheritance or relatively recent horizontal transfer of larger genomic regions. The densely interconnected clusters within the network analysis, strongly supported by a strikingly conserved syntenic architecture across the majority of isolates, indicate that the *fimD* adhesin gene is a stable genomic component propagated primarily through clonal expansion. This high level of conservation suggests that *fimD* is a core virulence determinant that is vertically inherited within successful and potentially hypervirulent *Klebsiella* lineages, rather than being frequently exchanged as a mobile element. The conserved synteny suggests a potentially important role for this gene within the overall fitness and virulence of certain *Klebsiella* clades.

The integrated phylogenetic, network, and synteny examinations elucidate a sophisticated interaction in the evolutionary mechanisms of the four supplementary genes (Figure 7A, Figure 8A, Figure 9A and Figure 10A) (Figure 7B, Figure 8B, Figure 9B and Figure 10B). Synteny analysis (Figure 7C, Figure 8C, Figure 9C and Figure 10C) corroborates this, demonstrating highly variable genomic contexts for *ycgR*, moderate conservation for *bamA*, some conservation for *sasA*, and strong conservation for *fimD*, indicating diverse acquisition mechanisms ranging from independent horizontal transfer to clonal expansion and vertical inheritance. Thus, the data highlight the diverse evolutionary forces shaping accessory gene distribution in *Klebsiella*, with horizontal gene transfer playing a particularly significant role in some genes but less so in others.

All of these findings point to *K. pneumoniae* in Hong Kong’s wet markets as genetically diverse pathogens that are resistant and persistent, which is alarming. We interpret these results in the discussion in light of the larger picture of microbial adaptation and public health.

## 4. Discussion

This investigation offers unprecedented genomic perspectives regarding the heterogeneity and acclimatization of *Klebsiella pneumoniae* strains derived from Hong Kong’s aquaculture markets, bearing significant ramifications for alimentary safety and communal health. Our analysis of pan-genome profiling, phylogenetic reconstruction, PCA, and antimicrobial resistance genes clarifies the evolution and transmission of *K. pneumoniae* in the environment.

**Phylogenetic Relationships and Potential Transmission Routes:** Phylogenetic analysis based on core-genome SNPs reveals a complex relationship between the wet market isolates and other *K. pneumoniae* strains. The wet market segregates do not group solely, signifying numerous contamination origins for the humid market. The absence of a unique wet market clade implies that the pollution is not confined to a solitary origin or transmission occurrence. A number of wet market isolates show parallel genetic links to clinical isolates, raising concerns about potential cross-contamination between these surroundings. This likely transfer accentuates the imperative of instituting rigorous hygiene protocols in both settings to alleviate the threat of propagating potentially pathogenic variants. A recent investigation elucidated considerable cross-contamination of pathobionts between aquaculture markets and industrial processing settings, suggesting potential contamination hazards [3]. The observation that animal *K. pneumoniae* isolates (particularly poultry and swine) form relatively tight clusters suggests transmission pathways within agricultural settings. The dispersal of environmental and surface isolates reflects the inherent diversity of microbial communities in the environment, including sewage and water. The diverse sources mentioned highlight the need for a more comprehensive approach to understand the epidemiology and transmission of *Klebsiella pneumoniae* within a broader ecosystem context.

**Principal Component Analysis (PCA):** The observed clustering patterns in both PCA plots (based on broad and specific sample types) align well with the phylogenetic analysis and further reinforce the hypothesis of distinct sources of contamination. The notable separation of wet market isolates in both PCA plots indicates potential for unique genomic adaptations to this environment. The observed clustering suggests that certain genetic factors might be associated with adaptation to specific ecological niches and host organisms. A recent study on the whole-genome sequencing of *K. pneumoniae* ST101 strains from various sources revealed distinct genetic signatures linked to host adaptation [32]. Hospital-associated strains, in particular, demonstrated higher levels of resistance and virulence compared to those from livestock, indicating adaptation to a specific host. The role of mobile genetic components (MGEs) in disseminating resistance genes was also emphasized by a comparative genomic examination of *K. pneumoniae* CC147 strains [33]. The dynamic nature of MGEs contributes to genetic diversity and adaptation across various environments. These studies highlight genetic factors in *K. pneumoniae* adaptation and reveal its evolutionary complexities. The overlapping regions in the finer-grained PCA plot (Figure 2B) highlight potential transmission routes and warrant further investigation, potentially employing advanced epidemiological methods to investigate the transmission routes between animal sources, sewage, and cutting boards.

**Pan-genome Architecture and Genetic Diversity:** The observation of an open pan-genome structure in our analysis of 43 *K. pneumoniae* genomes, including 4 isolates from Hong Kong wet markets, underscores the remarkable genetic diversity within this species. The persistent escalation of accessory genes, even subsequent to the incorporation of a considerable number of genomes, underscores the potential for continued horizontal gene transfer (HGT) and adaptation to various ecological niches. This plasticity is expected in a species like *K. pneumoniae*, known for its capacity to thrive in various environments, ranging from clinical settings to diverse environmental reservoirs [34]. The identification of a significant proportion of strain-specific genes (35.8%) further emphasizes the substantial genomic heterogeneity among these isolates. The presence of unique genes in three of the four wet market isolates (AL202S, SW202T3, and SF201T1) suggests potential adaptations to the specific environmental pressures encountered in wet markets, including the selective pressures exerted by cleaning agents, disinfectants, or the complex interplay of microbial communities within biofilms on wooden cutting boards. A recent study showed that *K. pneumoniae* Kp13 has significant genome plasticity, with 13 unique regions and specific plasmid genes [35]. This adaptability enables it to thrive in clinical and environmental niches. Research indicates that genomic rearrangements and recombination enhance the evolution of *K. pneumoniae*, increasing its virulence and resistance and allowing for survival across diverse environmental contexts [35]. The absence of unique genes in SF201T2 warrants further investigation to determine whether this isolate represents a less-adapted or recently introduced strain. The relatively high number of unique genes found in AL202S (183) compared to the other wet market isolates implies potential for a specific adaptation to this unique wet market setting.

**Antimicrobial Resistance Genes (ARGs):** The detection of a wide array of ARGs in the wet market isolates, especially the high ARG count in AL202S, presents a significant public health concern. The significance of antibiotic efflux as a key mechanism of multidrug resistance in *K. pneumoniae* from wet markets is highlighted by the prevalence of efflux pump genes in all four isolates. The proficiency of microorganisms to discharge a varied spectrum of antimicrobial compounds, which cultivates resistance, is primarily dependent on efflux mechanisms, especially those delineated by the *oqxAB* and *acrAB* genetic sequences [36]. This system presents considerable worries, as it may lead to resistance against various treatment alternatives, thereby complicating management approaches. This raises concern about the potential for widespread dissemination of antibiotic resistance from wet markets into the broader community. The presence of genes associated with modifying antibiotic targets and antibiotic inactivation (like *FosA6*) underscores the complexity and multi-faceted nature of antibiotic resistance mechanisms in these strains. The unique ARG profiles of each isolate suggest diverse acquisition pathways, highlighting the dynamism of HGT within these environments. The consistent presence of *acrB* across all isolates may indicate a common ancestral lineage or a highly successful HGT event involving this crucial RND efflux pump. Efflux pumps are a major resistance mechanism, but mutations in antibiotic targets and beta-lactamase genes also contribute to the MDR phenotype of *K. pneumoniae* [37]. The interplay of these factors, in conjunction with the activity of efflux pumps, establishes a complex resistance landscape that necessitates comprehensive strategies for effective management.

### 4.1. Virulence Factors in the Wet Market Samples

Biofilm-associated virulence factors in *Klebsiella pneumoniae* obtained from wet markets signify their pathogenicity. Biofilms help the bacterium resist immune responses and antimicrobial treatments, complicating infection management [38]. This attribute significantly enhances *K. pneumoniae*’s pathogenic potential when combined with supplementary virulence factors. The dynamic nature of bacterial evolution and the emergence of hypervirulent strains highlight the ongoing challenge in managing infections caused by *K. pneumoniae* in both clinical and food processing environments, necessitating continuous research and development of novel therapeutic approaches and optimized surface hygiene strategies.

*Klebsiella pneumoniae* utilizes a variety of intricate strategies and approaches to expertly acquire essential iron, which is crucial in bolstering its pathogenic capabilities by promoting both its proliferation and the formation of biofilms that assist in its endurance and virulence across various settings. These encompass the synthesis of siderophores, altering the functionality of the host’s iron metabolic pathways, and employing vesicles within the external membranes [39,40]. Iron availability significantly affects biofilm formation in *K. pneumoniae*, with lower iron concentrations promoting denser biofilm structures. This is mediated by metabolic changes, such as the suppression of succinic acid [41]. Such features associated with wet market *K. pneumoniae* further suggest their potential pathogenicity.

The capsulated phenotype of wet market *K. pneumoniae* strains is a significant virulence factor that can enhance other virulence traits, including iron acquisition [42]. The capsule’s protective role extends to safeguarding outer membrane receptors and iron-binding proteins, which are crucial for iron uptake, a vital process for bacterial survival and pathogenicity [43]. This enhancement of iron acquisition is part of a broader strategy that includes other virulence factors, such as biofilm formation and hypermucoviscosity, which collectively contribute to the bacterium’s fitness and virulence [44].

The observed variability in the virulence gene profiles of *Klebsiella pneumoniae* isolates from wet markets can be attributed to potential contamination from clinical sources and the bacterium’s high genome plasticity, facilitated by horizontal gene transfer [45]. This variability indicates a complex interaction between environmental and clinical strains, potentially leading to more virulent and antibiotic-resistant ones. *K. pneumoniae*’s genomic flexibility is key to its adaptability and pathogenicity, shown by the variety of virulence and resistance genes in different isolates [46]. The variation in virulence gene profiles emphasizes *K. pneumoniae*’s adaptability while also revealing the difficulties in managing its spread. The risk of horizontal gene transfer and contamination from clinical environments calls for strong surveillance and control strategies to stop the increase in highly virulent and resistant strains in both clinical and environmental contexts.

### 4.2. Accessory Genes: Evolutionary Dynamics and Horizontal Gene Transfer

The combined phylogenetic, network, and synteny analyses of four selected accessory genes (*ycgR*, *sasA*, *bamA*, and *fimD*) reveal distinct evolutionary trajectories influenced by both vertical inheritance and HGT. The highly variable genomic context observed for *ycgR* strongly supports independent HGT events, whereas the relatively conserved synteny around *fimD* indicates primarily vertical inheritance. The intermediate patterns for *sasA* and *bamA* suggest a complex interplay between both vertical inheritance and HGT in shaping their distribution among isolates. The highly dispersed network topology for *ycgR*, coupled with its highly variable genomic context, strongly suggests the involvement of mobile genetic elements in its dissemination, potentially via plasmids or transposons. The denser clustering observed in the network for *fimD* suggests a predominantly clonal spread, highlighting the role of vertical inheritance in maintaining the prevalence of *fimD* in certain lineages. The network visualizations provide complementary evidence for the conclusions drawn from the phylogenetic analysis, clearly distinguishing the roles of vertical inheritance and horizontal gene transfer for these selected accessory genes. The diversity in evolutionary mechanisms among accessory genes in *Klebsiella pneumoniae* underscores the complex evolutionary processes that shape its genome across various environments. Numerous determinants affect this heterogeneity, encompassing plasmid recombination, lateral gene transfer, and selective stresses from diverse ecological habitats [34,47]. The complex processes within *K. pneumoniae* enhance its ability to become more adaptable and dangerously resilient, especially in specific environments where antimicrobial agents fail to work effectively or do not deliver their intended therapeutic effects [47]. The flexible mechanisms discerned in *K. pneumoniae* are distinguished by their heterogeneity and intricacy, yet they are not solely distinctive to this species of organism. Similar processes are observed in other bacterial pathogens, where horizontal gene transfer and plasmid dynamics also play pivotal roles in shaping genomic diversity and adaptability [48]. An understanding of the mechanisms in *K. pneumoniae* can yield insights into broader bacterial evolution and inform strategies for the management of antibiotic resistance.

### 4.3. Integrated Results

Through the integration of the collective findings from our analyses, it becomes apparent that the *K. pneumoniae* isolates originating from wet markets in Hong Kong constitute a genetically diverse population, exhibiting varying degrees of connection to clinical, environmental, and animal isolates. This diversity is likely driven by a combination of factors, including multiple contamination sources and ongoing HGT events facilitated by the complex ecosystem of wet markets. The presence of ARGs in a significant proportion of the isolates underscores the potential for antimicrobial resistance dissemination from these environments to other settings. The diverse evolutionary mechanisms shaping the accessory genome, encompassing both vertical inheritance and HGT, further highlight the dynamic interplay of evolutionary forces within the wet market environment. The identification of specific accessory genes with distinct evolutionary patterns offers potential targets for future studies aimed at comprehending the adaptive strategies of *K. pneumoniae* in wet markets.

While our analysis offers the first genomic insights into *K. pneumoniae* from the wet markets of Hong Kong, it is imperative to consider several limitations. The limited sample size (*n* = 4 wet market isolates) constrains inferences regarding strain prevalence in these surroundings. However, the inclusion of globally representative public genomes (e.g., ST101/ST147) contextualizes our findings within broader antimicrobial resistance (AMR) dissemination patterns. Of particular concern, the detection of clinical-associated ST258 in wet markets underscores the urgent need for integrated surveillance across human–animal–environment interfaces, as advocated by the One Health framework. These genomic data could directly inform targeted interventions: high-risk stalls exhibiting clinical strain linkages could prioritize replacing wooden cutting boards with non-porous materials and implement enhanced disinfectant protocols, specifically targeting AMR gene reservoirs (*blaCTX-M*, *oqxAB*). Such measures would disrupt transmission networks while maintaining market functionality.

Although we provide valuable insights with our observations, the small number of wet market isolates (*n* = 4) does not allow for conclusions to be made with certainty regarding the prevalence of strains. In addition, the predictive value of virulence and resistance based on genomes should be confirmed by phenotyping, which is the center of our ongoing investigation.

### 4.4. Limitations

Three limitations warrant consideration. First, the small sample size of wet market isolates (*n* = 4) limits statistical power to assess gene frequency differences across ecological niches (e.g., clinical vs. environmental). While our inclusion of 39 public genomes contextualizes diversity, larger-scale sampling of wet markets is needed to infer strain prevalence or niche-specific adaptations. Second, despite curating public genomes for ecological representation, database biases toward clinically relevant sequence types (e.g., ST258, ST11) may skew phylogenetic inferences about environmental transmission routes. Third, while genomic predictions highlight putative virulence and resistance traits (e.g., *fimD*-mediated biofilm formation, *acrB*-driven efflux), phenotypic validation through microbiological assays (e.g., biofilm quantification, antibiotic susceptibility testing) is critical to confirm functional implications. Future studies should integrate expanded wet market sampling with metagenomics to resolve community-level horizontal gene transfer (HGT) dynamics and niche-specific selection pressures.

## 5. Conclusions

This genomic research provides essential evidence that the wet markets in Hong Kong act as reservoirs for genetically diverse and potentially dangerous strains of *Klebsiella pneumoniae*. A troubling pathway for pathogen transmission was highlighted by our analysis, which showed that isolates from this environment are not just environmental saprophytes but also exhibit strong phylogenetic ties to high-risk clinical clones. Most importantly, these strains have a strong set of virulence determinants that promote biofilm formation and persistence on market surfaces, as well as a powerful arsenal of antimicrobial resistance genes, most of which encode efflux pumps that confer multidrug resistance. These populations’ exceptional capacity for adaptation, which allows them to flourish in the face of the intricate ecosystem’s selective pressures, is demonstrated by their open pan-genome structure and evidence of widespread horizontal gene transfer. These results change the perception of wet markets as merely locations where food deteriorates to one that acknowledges them as possible sources of antibiotic resistance in the urban food chain. Therefore, there is a pressing need to incorporate wet markets into a One Health surveillance system and to adopt improved, evidence-based hygiene practices, such as replacing porous cutting surfaces and using disinfectants that are effective against biofilms. Despite the small sample size, this study offers the first high-resolution genomic proof of this serious public health concern, highlighting the need for more extensive epidemiological and metagenomic research to measure the frequency and dynamics of transmission of multidrug-resistant pathogens in these crucial but vulnerable food hubs.

## Figures and Tables

**Table 1:** Sample genome description.

Sample	Host	Type	BioSampleID
AL202S	Food_proc_surface	Wet market	SAMN45810452
SF201T1	Food_proc_surface	Wet market	SAMN45810453
SF201T2	Food_proc_surface	Wet market	SAMN45810454
SW202T3	Food_proc_surface	Wet market	SAMN45810455
GCA_013349065	Surface	Environmental	SAMN15186438
GCA_022354665	Sewage	Environmental	SAMN13925598
GCA_000240185	Homo-Sapiens	Clinical	SAMN02602959
GCA_013376555	Water	Environmental	SAMN14548317
GCA_022559765	Poultry	Animal-associated	SAMN23139062
GCA_001887985	Homo-Sapiens	Clinical	SAMN06019522
GCA_013423825	Sewage	Environmental	SAMN15511588
GCA_022559785	Poultry	Animal-associated	SAMN23139063
GCA_002164695	Homo-Sapiens	Clinical	SAMN06909168
GCA_014041505	Surface	Environmental	SAMN15649164
GCA_022749475	Swine	Animal-associated	SAMN26814915
GCA_002164855	Homo-Sapiens	Clinical	SAMN06909161
GCA_014041845	Homo-Sapiens	Clinical	SAMN15649146
GCA_023242905	Homo-Sapiens	Clinical	SAMN27593784
GCA_002852995	Homo-Sapiens	Clinical	SAMN07609113
GCA_014840955	Poultry	Animal-associated	SAMN16222891
GCA_023572965	Homo-Sapiens	Clinical	SAMN28546263
GCA_003261955	Homo-Sapiens	Clinical	SAMN08932863
GCA_015353095	Swine	Animal-associated	SAMN16659219
GCA_023650835	Sewage	Environmental	SAMN23799605
GCA_003432405	Swine	Animal-associated	SAMN09691066
GCA_017815795	Red_Kangaroo	Animal-associated	SAMN14590646
GCA_024299245	Homo-Sapiens	Clinical	SAMN26245098
GCA_006974165	Rabbit	Animal-associated	SAMN11650121
GCA_018449065	Swine	Animal-associated	SAMN19107302
GCA_025152765	Homo-Sapiens	Clinical	SAMN30672525
GCA_008632415	Poultry	Animal-associated	SAMN12741260
GCA_018487825	Swine	Animal-associated	SAMN12741260
GCA_026821715	Homo-Sapiens	Clinical	SAMN30914361
GCA_013348885	Homo-Sapiens	Clinical	SAMN15186440
GCA_019334625	Swine	Animal-associated	SAMN20245095
GCA_013348905	Surface	Environmental	SAMN15186447
GCA_020990405	Housefly	Animal-associated	SAMN23139350
GCA_013348985	Surface	Environmental	SAMN15186444
GCA_021496545	Swine	Animal-associated	SAMN15097741
GCA_013349005	Surface	Environmental	SAMN15186443
GCA_022354125	Sewage	Environmental	SAMN13925581
GCA_013349025	Surface	Environmental	SAMN15186442
GCA_022354365	Sewage	Environmental	SAMN13925526

## Data Availability

The datasets analyzed in this study are available in the NCBI SRA (Sequence Read Archive) under the following BioSample accession numbers: SAMN45810452; SAMN45810453; SAMN45810454; SAMN45810455; SAMN15186438; SAMN13925598; SAMN02602959; SAMN14548317; SAMN23139062; SAMN06019522; SAMN15511588; SAMN23139063; SAMN06909168; SAMN15649164; SAMN26814915; SAMN06909161; SAMN15649146; SAMN27593784; SAMN07609113; SAMN16222891; SAMN28546263; SAMN08932863; SAMN16659219; SAMN23799605; SAMN09691066; SAMN14590646; SAMN26245098; SAMN11650121; SAMN19107302; SAMN30672525; SAMN12741260; SAMN12741260; SAMN30914361; SAMN15186440; SAMN20245095; SAMN15186447; SAMN23139350; SAMN15186444; SAMN15097741; SAMN15186443; SAMN13925581; SAMN15186442; SAMN13925526. The data can be accessed through the NCBI SRA portal at https://www.ncbi.nlm.nih.gov/sra/ (accessed on 7 August 2025).
